# The cost-effectiveness of food consistency modification with xanthan gum-based Nutilis Clear® in patients with post-stroke dysphagia in Poland

**DOI:** 10.1186/s12913-020-05411-2

**Published:** 2020-06-17

**Authors:** Aleksandra Pelczarska, Michał Jakubczyk, Maciej Niewada

**Affiliations:** 1 HealthQuest, Warsaw, Poland; 2 Decision Analysis and Support Unit, SGH Warsaw School of Economics, Warsaw, Poland; 3 Department of Experimental and Clinical Pharmacology, Medical University of Warsaw, Warsaw, Poland

**Keywords:** Dysphagia, Stroke, Aspiration pneumonia, GUSS scale, Food consistency, Nutilis clear, Xanthan gum

## Abstract

**Background:**

Dysphagia is a well-known stroke complication characterised by difficulty in swallowing. It may affect the majority of stroke patients and increases mortality and morbidity, due to aspiration pneumonia and malnutrition. Food thickening may help patients to feed themselves, and its effectiveness was demonstrated. However, the cost-effectiveness studies are lacking. We evaluate the cost-utility of xanthan gum-based consistency modification therapy (Nutilis Clear®) in adult post-stroke patients from the public payer perspective in Poland.

**Methods:**

Routine clinical practice was used as a comparator, as no alternative specific treatment for dysphagia is available. To verify the robustness of the results against the modelling approach, we built two models: a static (a fixed simple-equations model, 8-week time horizon of dysphagia) and a dynamic one (Markov model, with a possible dysphagia resolution over a 1-year horizon). In both models, the treatment costs, health state utilities, and clinical events (i.e. aspiration, aspiration pneumonia, death) were included. Parameters were estimated jointly for both models, except for the duration of dysphagia and the risk of aspiration pneumonia (specific to the time horizon). We only assumed Nutilis Clear® to prevent aspirations, without affecting dysphagia duration.

**Results:**

The average cost of one quality-adjusted life year (i.e. the incremental cost-utility ratios, ICURs) amounted to 21,387 PLN (€1 ≈ 4.5 PLN), and 20,977 PLN in static and dynamic model, respectively; far below the cost-effectiveness threshold in Poland (147,024 PLN). The one-way, scenario, and probabilistic sensitivity analysis confirmed these findings.

**Conclusions:**

Nutilis Clear® is highly cost-effective in Poland from the public payer perspective. Our approach can be used in other countries to study the cost-effectiveness of food thickening in stroke patients.

## Background

Dysphagia affects 37–78% stroke patients, depending on the diagnostic method [1]. Dysphagia may be associated with increased mortality and morbidity due to aspiration pneumonia and malnutrition [2–6] as well as decreased quality of life [7–9]. It is rarely an isolated health problem, and despite many advances in healthcare post-stroke dysphagia remains unappreciated [10].

Food consistency modification may help patients with dysphagia to feed themselves and, as a result, it may lead to improved clinical outcomes [11]. For that purpose different products are available, with xanthan gum and modified starch-based products being the most common. The effectiveness of food thickening was shown in stroke patients [12] and other populations [13]. However, the attempts to evaluate the cost-effectiveness of such a treatment have been limited. At the same time, such an evaluation could inform about the clinical and economic consequences of financing the food modification from public money and result in optimizing the care for post-stroke patients.

In the present paper, we evaluate the cost-utility of xanthan gum-based consistency modification therapy (Nutilis Clear®) in adult stroke patients with dysphagia from the public payer perspective in Poland. Routine clinical practice (henceforth, RCP) was used as a comparator, as no alternative specific treatment for dysphagia was available. RCP consists of the use of behavioural compensations and manoeuvres (posture, sensory enhancement) as well as rehabilitation exercises aiming to alter swallowing physiology through strength and skill exercises.

The contribution of the present paper is two-fold. Firstly, we present the first (to the best of our knowledge) economic model allowing the cost-utility assessment of consistency modifiers in patients with post-stroke dysphagia. Secondly, we specifically assess one such technology in Poland to inform the rationale for its reimbursement. The technology in question was recently recommended for reimbursement by the Polish Agency for Health Technology Assessment and Tariff System (AOTMiT), also based on the results of the present model [14, 15]. The model can be used in other countries to inform decisions on care provided for stroke patients.

All stroke patients should be screened for dysphagia as soon as possible. In Poland, the access to instrumental screening methods is limited owing to high costs. Therefore, we restricted our analysis to patients with the aspiration level 10–14 on Gugging Swallowing Screen (GUSS) scale (patients who tolerate semisolid intake but not fluids) recommended by local guidelines [16]. The GUSS scale involves direct swallowing test with different food consistencies, it is characterized by good sensitivity, acceptable specificity, and can be performed in all stroke patients [17]. Its results were recently successfully revalidated [18]. GUSS scale is referred to in Polish guidelines for nutritional treatment in neurology [19]. Population of adult stroke patients with dysphagia and aspiration of 10–14 on GUSS scale was estimated to be around 7500 patients a year in Poland based on yearly number of strokes and dysphagia and aspiration prevalence in stroke patients [18, 20, 21].

## Methods

### General approach

**Fig. 1:**
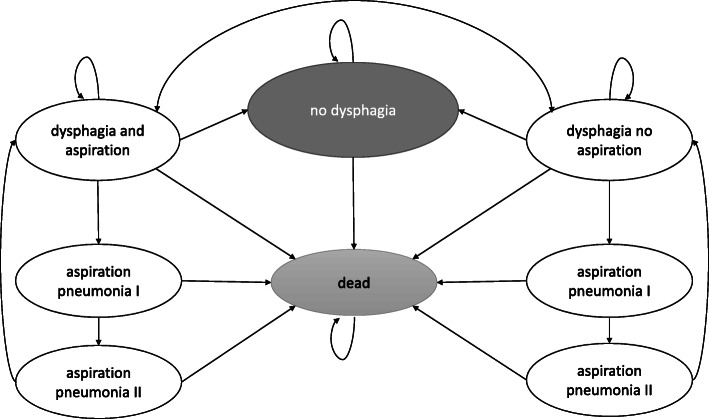
Dynamic model structure (*API and APII stands for aspirating pneumonia 1st and 2nd week of treatment, respectively)

To verify the robustness of the results against the approach used, we built two models: a static and a dynamic one. In the former, a fixed duration of dysphagia is assumed (8 weeks, based on [22]); this approach serves to approximate the cost-utility with simple equations and a minimal set of assumptions, even if in a rather simplified setting (see Table 1, for details). In the latter, to reflect a possible dysphagia resolution process, we built a Markov model with patient transitions between health-states within a one-year horizon; this approach serves to represent the actual clinical process better, yet at the expense of increasing the number of assumptions and parameters to be estimated. Figure 1 illustrates the structure of the model. Both models were implemented in MS Excel.

**Table 1:** Static model structure

Category	Equations
**Intervention arm**	
Costs	C_intervention_ = C_Nutilis Clear_(8 weeks) + [C_aspiration pneumonia_ ∙ *p*(aspiration penumonia| non − aspirators)]
Outcomes	QALY_intervention_ = [U_no aspiration_ ∙ 8/52] − [*∆*U_aspiration pneumonia_ ∙ 2/52 ∙ *p*(aspiration pneumonia| non − aspirators)]
**Comparator arm**	
Costs	C_comparator_ = [C_aspiration pneumonia_ ∙ *p*(aspiration pneumonia| aspirators)]
Outcomes	QALY_comparator_ = [*U*_*aspiration*_ ∙ 8/52] − [*∆*U_aspiration pneumonia_ ∙ 2/52 ∙ *p*(aspiration pneumonia| aspirators)]

*Abbreviations*: *C* cost, *QALY* quality-adjusted life-year, *p* probability, *U* utility

In both models, the treatment costs, health state utilities, and the risk of clinical events (i.e. aspiration in patients with dysphagia, aspiration pneumonia, death) were included. Parameters were estimated simultaneously, except for the duration of dysphagia and the probability of aspiration pneumonia, which was specific to the time horizon of the analysis. In both models, we assumed Nutilis Clear® does not affect the dysphagia duration but reduces the risk of aspiration pneumonia (by preventing aspirations, as reported by [21, 23], see also Table 1 in the Supplementary Material). When setting models’ parameters, we used a literature review and consulted clinical experts to validate the final values used; Polish data was preferred. Two stage process was employed: the proposed parameters values were first consulted in the form of structured survey and secondly discussed to consensus. Populating the model is described below.

### Clinical parameters

#### Probability of dysphagia resolution

In the dynamic model, the dysphagia resolution was modelled based on the natural course of dysphagia in stroke patients. The number of patients experiencing dysphagia 0/7/28/180 days after stroke was extracted [24, 25], and we assumed no patients have dysphagia after one year; then, the weekly probability of dysphagia resolution in the first year was calibrated to fit the data, assuming linearity (see also Tables 2 and 3 in the Supplementary Material).

#### Probability of aspiration pneumonia and treatment duration

The duration of aspiration pneumonia was set to two weeks, based on the time of antibiotic therapy [26], i.e. approx. Ten days, with additional four days of a decreased health utility due to recent disease symptoms. This assumption was confirmed by the clinical experts.

In the static model, the risk of aspiration pneumonia amounted to 12.2% (10 out of 82) and 1.75% (1 out of 57) in patients with/without dysphagia, respectively (based on [27], a study with a similar follow-up time to the present analysis horizon).

In the dynamic model, the risk of aspiration pneumonia was estimated in longer follow-up (1-year horizon) in several steps. First the risk of aspiration pneumonia in patients without post-stroke dysphagia was estimated based on published data identified in literature review (based on the number of patients who developed aspiration pneumonia [27–31], data for 264 patients in total). The overall risk and the weekly incidence rate ($\mathrm{IR}=\frac{\left[-\ln \left(1-\mathrm{CR}\right)\right]}{\mathrm{T}\ \left[\mathrm{weeks}\right]}$, CR = cumulative risk of event in study follow-up, T = weeks of follow-up) were estimated for each study. Finally, the averaged (weighted by the number of patients) incidence rate amounts to 0.4036%; 95%CI: 0.00; 2.56; for raw data see also Table 4 in the Supplementary Material).

Then, to estimate the corresponding risk for patients with post-stroke dysphagia, we used the relative risk (RR) based on number of patients who developed pneumonia in the group of patients with dysphagia in general (regardless the cause of dysphagia). Estimations were based on aspiration pneumonia prevalence in patients with dysphagia and with (24 out of 83) or without aspirations (15 out of 155) in comparison to patients without dysphagia (5 out of 143), 28.9 and 9.7% vs 3.5%, respectively [32]. Eventually, RR of aspiration pneumonia in patients with dysphagia but without aspirations was calculated to 2.77 (95%CI: 1.03; 7.42) and RR of aspiration pneumonia in patients with dysphagia and aspirations to 8.27 (95%CI: 3.28; 20.85).

#### Probability of death

Higher risk of death in the first 30 and 90 days post stroke (hospital mortality) was estimated to 4.60 and 0.96% weekly, respectively. The risk of death in subsequent weeks was estimated to 0.24% (up to 52nd week). Estimations were based on the annual stroke mortality (stratified by stroke subtype) in Poland [33] weighted by stroke subtype incidence in Poland [34, 35]. The weighted average of stroke mortality was estimated and assumed to be constant for subsequent time intervals. Eventually, weekly estimates were calculated (for raw data see Tables 5 and 6 in the Supplementary Material,). Different parameter values were modelled and tested in the sensitivity analysis (SA) based on time-dependent risk estimation. Data on cumulative stroke mortality (30, 90, 180, and 360 days’ post-stroke) of 269 stroke patients hospitalized in the Regional Hospital in Krosno, Poland in 2003/2004 were used. The data was interpolated using a logarithmic function with a very high reproduction of experimental points (R^2^ = 0.9947) and the risk of death in subsequent weeks was determined over a one-year period (for raw data see also Table 7 in the Supplementary Material).

Aspiration pneumonia is associated with an increased risk of death in comparison to patients without aspiration pneumonia (RR = 2.99; 95%CI: 2.44; 3.66; as estimated by [36]).

#### Probability of dysphagia deterioration

The probability of dysphagia symptoms exacerbation over time, i.e. the start of aspirations, was estimated to 0.11% weekly (3 of 120 patients in a 6-month follow-up [28];).

#### Monitoring

In line with the European guidelines for the diagnosis and treatment of dysphagia for stroke patients [37], the re-assessment of patients’ condition should be performed within one week and maximum every 2–3 months thereafter within the first year. In the model, we assumed the monitoring takes place in weeks: 1, 4, 12, 26, 39, and 52 (experts’ opinion).

### Health states utilities

In the static model, the following health states were considered: dysphagia with/without aspiration and aspiration pneumonia. In the dynamic model, we included the following states: no dysphagia, dysphagia with/without aspiration, aspiration pneumonia in aspirating patients, aspiration pneumonia in non-aspirating patients, and death. To estimate the utility of the health states, a systematic review of literature was carried out (see also Section 1.3 in the Supplementary Material). All values were subsequently consulted with clinical experts to assess face validity.

#### Utilities related to dysphagia

Direct utilities values as well as relative utilities (disutilities) were used in the model [38]. estimated the average health state utility in 430 patients with post-stroke dysphagia and delayed enteral nutrition recommendation using EuroQoL questionnaire (based on publication data, 2005, likely EQ-5D-3 L questionnaire was used). As no direct utility values for patients with dysphagia and aspirations were identified, the data from [38] were used for approximation. We assumed population of patients with delayed enteral nutrition would be the most comparable to population fed orally with the use of Nutilis Clear®. The assumptions that the two health states should be similar enough in terms of quality of life was confirmed by clinical experts.

Obviously, patients’ health state and subsequently associated health state utility change over time. In the dynamic model (with 1-year follow-up), this is represented by transition between health states not by change in health state utility itself (we assumed that each health state in the model is represented by specified health state utility). Data were collected by [38] in one-time point (after 6 month of follow-up) and approximate one health state. We expect that condition of patients with post-stroke dysphagia improves over time and the value of utility may be overestimated in our model. Thus, the assumption we made is conservative (a high one) and reduces the cost-effectiveness of the intervention under study. The estimation uncertainty was not reported in the original paper; thus, we assumed 10% parameter value change (0.135; 0.165) in SA (one-way deterministic and probabilistic). Beta distribution was used in PSA and backward calculation of standard deviation (SD) was performed based on the confidence interval.

Patients with no aspirations rarely choke on food or cough and have voice changes less frequently [12, 23]. Therefore, the treatment resulting in absence of aspirations improves the quality of life [39] and increases the utility.

Due to the lack of specific data for patients with post-stroke dysphagia without aspirations, we considered head or neck cancer patients with dysphagia. The incremental values, i.e. the utility value of the health condition gained due to the removal of the patient’s aspiration were estimated as follows [40]. evaluated the severity of dysphagia (*N* = 50 patients) according to the point classification: 0 = no dysphagia/aspiration; 1 = dysphagia when eating solid foods; 2 = dysphagia when eating semi-solid (pasty) food; 3 = dysphagia with fluid intake; and 4 = dysphagia/aspiration of saliva. As the use of Nutilis Clear® is associated with the removal of aspiration on fluids, we assumed the utility increase can be approximated by the difference between level 3 and level 0. Thus, the utility of dysphagia without aspiration was estimated at 0.37 (i.e., the sum of the utilities of the condition for patients with dysphagia and aspiration, which was 0.15 plus incremental utility value associated with avoiding aspiration, which was estimated at 0.22 – increment between level 3 and level 0). In SA (one-way deterministic and probabilistic) confidence interval was used based on parameter beta distribution (95%CI: 0.32; 0.42), backward calculation of SD was performed based on the confidence interval.

#### Utilities related to aspiration pneumonia

We did not identify any quality-of-life study in stroke patients with aspiration pneumonia. Therefore, we used data for > 65 years old patients with community-acquired pneumonia (*N* = 562), obtaining the disutility equal to 0.13 [41]. In the SA, the maximum and minimum value of the decrement reported in [41] was tested, 0.15 and 0.10, respectively (a conservative assumption, i.e. a low one, in experts’ view).

### Costs

We included direct costs of dysphagia treatment (including Nutilis Clear® costs), aspiration pneumonia treatment, and monitoring. The latter one was included in dynamic model only. Monitoring was omitted in static model to maintain minimal number of assumptions. Static model has relatively short horizon (8 weeks) resulting in maximum two monitoring visits and thus having minimal impact on model results.

We assumed Nutilis Clear® (175 g re-sealable tin) is available with a patients flat-rate payment (3.20 PLN) and costs 77.05 PLN from NFZ perspective (€1 ≈ 4.5 PLN). The average daily consumption of Nutilis Clear® was calculated based on share of each available product consistency [42], and the average daily demand for fluids in post-stroke patients (9 cups [43];). Weighted mean daily product consumption was estimated to 37.96 g and was constant over time, see also Table 8 in the Supplementary Material.

The cost of monitoring and aspiration pneumonia treatment were calculated based on Polish NFZ data and was estimated to 186 PLN and 1924 PLN, respectively. Both costs were constant over time. This reflects to Polish healthcare funding system where pricing is based on Diagnosis Related Group pricing (fully covered by public payer).

### Validation

Rigorous validation process was incorporated to determine results’ applicability and model transparency. This involved consultation of model parameters with clinical experts. Subsequently, model was systematically tested to identify errors related to data introduction and the model structure. Systematic literature search did not show any published models focused on the same decision problem. Thus, two economic were built using different modelling approach to provide convergence validation. The extent of external and predictive validation was limited due to lack of published registry studies covering health state utility assessment in patients with post-stroke dysphagia. However, many parameters were time-dependent and sourced from long-term trials (i.e. dysphagia resolution and risk of mortality). As many models, this model was built to synthesize the best available evidence and illuminate a policy decision for which no trial is ongoing.

### Sensitivity analysis

Different scenarios of SA (deterministic one-way and multi-way, probabilistic) were performed. One-way deterministic sensitivity analysis covered parameters with the greatest uncertainty. Both models were tested with alternative values of: the risk of aspiration pneumonia in patients with/without aspirations, the utility of having aspirations, the utility decrement of aspiration pneumonia occurrence. Dynamic model (due to having more parameters) was tested with alternative values of: the risk of aspiration pneumonia in patients without post-stroke dysphagia, the relative risk of aspirations pneumonia in patients with dysphagia but without aspirations, the relative risk of aspiration pneumonia in patients with dysphagia and aspirations, the relative risk of death in post-stroke patients with aspiration pneumonia, and the utilities of no dysphagia, dysphagia with aspirations, aspiration pneumonia (decrement). Additionally several scenario (deterministic multiway) sensitivity analysis were performed testing alternative consumption of Nutilis Clear® patterns, costs of aspiration pneumonia treatment (both models) as well as impact of false positive diagnosis on GUSS screening test, lifetime analysis horizon, lack of monitoring, risk of death values (based on [34]) and cost effectiveness estimation where effectiveness of Nutilis Clear® stems only from the patients’ years of life gained (dynamic model). On top of that, probabilistic SA was implemented and executed (for detailed description see Section 1.4 in the Supplementary Material).

## Results

The incremental cost-utility ratios (ICURs), i.e. the cost of one additional quality-adjusted life year (QALY), were calculated for each model (Table 2). Therapy with Nutilis Clear® (in comparison to RCP) was associated with gain in QALY and additional costs to public payer in both models. ICUR amounted to 21,387 PLN and 20,977 PLN in static and dynamic model, respectively. Thus, ICUR was far below cost-effectiveness threshold in Poland (147,024 PLN, as of now). The biggest contribution to costs had the cost of Nutilis Clear®. The biggest savings were made due to reduction of aspiration pneumonia prevalence with Nutilis Clear®.

**Table 2:** Results of basic analysis vs RCP. Costs in PLN. ICUR in PLN/QALY – static and dynamic model

Category	Nutilis Clear®	RCP	Nutilis Clear® vs RCP
**Static model**			
Effects (QALY)	0.057	0.022	0.035
Total costs [PLN], incl	970	235	735
dysphagia treatment	936	0	936
aspiration pneumonia treatment	34	235	− 201
ICUR [PLN]	–	–	21,387
**Dynamic model**			
Effects (QALY)	0.351	0.331	0.020
Total costs [PLN], incl	994	570	424
dysphagia treatment	583	0	583
aspiration pneumonia treatment	83	243	− 160
Monitoring	329	327	2
ICUR [PLN]	–	–	20,977

*Abbreviations*: *ICUR* incremental cost-utility ratio, *QALY* quality-adjusted life-year, *PLN* Polish Zloty, *RCP* routine clinical practice

**Fig. 2:**
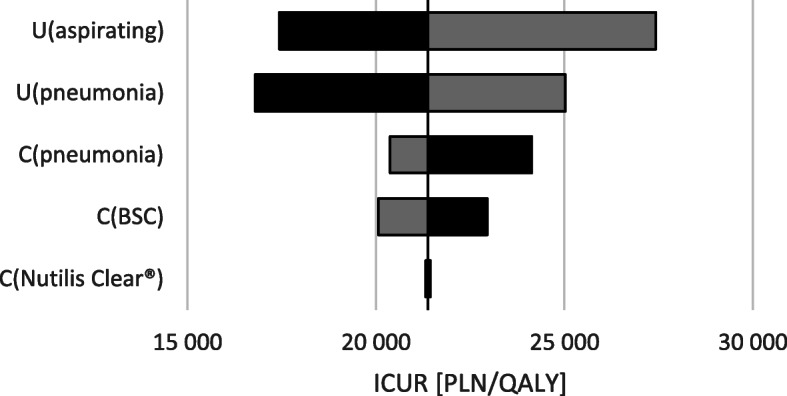
Results of a one-way sensitivity analysis vs. BSC for static model. (gray = minimum parameter value, black = maximum parameter value)

**Fig. 3:**
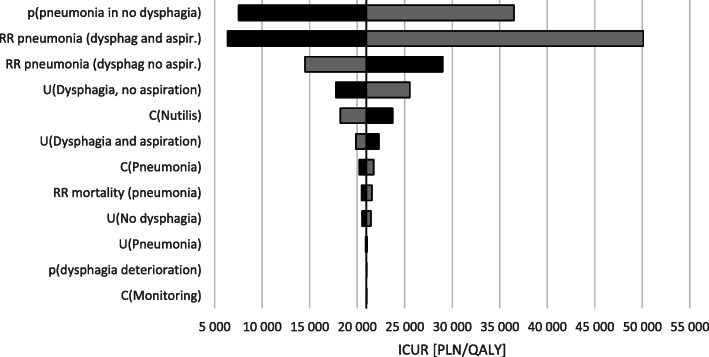
Results of a one-way sensitivity analysis vs. BSC. Dynamic model (gray = minimum parameter value, black = maximum parameter value)

The results of one-way and scenario sensitivity analyses showed that Nutilis Clear® therapy in patients with post-stroke dysphagia is cost-effective from the public payer perspective in all scenarios (max ICUR was below 77,389 PLN). The biggest impact on analysis results was associated with utility increment of aspirations removal in patients with post-stroke dysphagia (static model, Fig. 2) and RR of aspiration pneumonia occurrence value in patients with dysphagia and aspirations (dynamic model, Fig. 3). The probability that Nutilis Clear® is a cost-effective intervention exceeds 99% in both models (based on cost-effectiveness acceptability curves; see also Section 1.5 in the Supplementary Material).

## Discussion

We compared the cost-effectiveness of food consistency modification with Nutilis Clear® with RCP in stroke patients with dysphagia with two different modelling approaches. To the best of our knowledge, our analysis is the first cost-effectiveness analysis of the food consistency modification in patients with dysphagia.

A static model with fixed dysphagia duration and a dynamic model, where dysphagia duration was modelled, were proposed. The results of both models were very consistent. Nutilis Clear® was a cost-effective intervention in comparison to RCP in all tested scenarios, i.e. ICUR was much lower than accepted cost-effectiveness threshold for quality-adjusted life year in Poland. The biggest effects resulted from decrease of aspiration pneumonia prevalence due to reduction in aspirations occurrence while on Nutilis Clear®.

It is important to stress that both models yielded very similar results. This robustness of the ICURs against the modelling approach increases the credibility of the final estimates but also is reassuring in terms of assumptions adopted in individual models.

All peer-reviewed model parameters were consulted with experienced clinical experts. Utility values were adopted from various studies; it should be noted, however, that the selection of specific values was made based on the results of a systematic literature review. Adopted values did not differ between analysis arms (both models).

Our study is limited by the data availability, especially these regarding Polish population of patients with post-stroke dysphagia. Lack of data did not allow us to study the cost-utility of Nutilis Clear® in subpopulations, for example as defined by age and sex. The recovery pattern and the risk of infectious complications most certainly differ in stroke subgroups. However, the evidence in the literature is limited and mixed. For example, in [2] no statistically significant differences with respect to age and sex were found between patients with or without dysphagia (in a sample of 570). On the other hand, [44, 45] identified statistically significant differences in age between patients with and without post-stroke pneumonia (the significance of differences in sex depended on the form of analysis). In view of the above-mentioned limitations, we constructed a model referring to a hypothetical average patient. This does not change the fact that the inability of our model to address the question of cost-utility in specific subpopulations is a limitation of our study. How the results could differ in those subpopulations is not clear to us. On the one hand, larger baseline risk (e.g. in older patients) may create larger margins for clinical benefits and further improve cost-utility of food modification. On the other hand, larger baseline risk may result in worse overall prognosis and reduce the benefits of reducing dysphagia. We believe that the sensitivity analysis, especially the probabilistic one, at least partially covers the extent of possible variability of the results.

We lack specific data on regimen patterns in Poland. Thus, the dosing of Nutilis Clear® was based on the average proportion of a given level of food consistency in medical prescriptions in US. Stable dosage was kept during the analyses. However, it may be expected that in real world patients would accept foods of lower viscosity over time as their condition improves. This would improve overall cost-utility of food consistency modification.

Other limitations are related to the nature of Markov model, in particular the memorylessness of model: the probability of transition to any given health state depends only on current health state affiliation. Still, removing this assumption would make it necessary to use a much more detailed, and most likely unavailable, data to set the parameters of the model.

## Conclusions

Analysing the cost-effectiveness of food modifiers in post-stroke patients with dysphagia is possible via economic modelling. Based on the best available evidence, Nutilis Clear® therapy appears to be highly cost-effective in Poland from the perspective of public payer.

## Supplementary information

**Additional file 1.**

## Data Availability

The datasets used and/or analysed during the current study are available from the corresponding author on reasonable request.

## Abbreviations

C: Cost
CR: Cumulative risk
EUR: Euro
GUSS: Gugging Swallowing Screen
ICUR: Incremental cost-utility ratio
IR: Incidence rate
NFZ: National Health Fund in Poland (Narodowy Fundusz Zdrowia)
QALY: Quality adjusted life year
p: Probability
PLN: Polish Zloty
RR: Relative risk
SA: Sensitivity analysis
SD: Standard deviation
T: Time, duration
U: Utility
RCP: Routine clinical practice
