# Supplementary material for: The cost-effectiveness of food consistency modification with xanthan gum-based Nutilis Clear® in patients with post-stroke dysphagia in Poland
Source: BMC Health Serv Res. 2020 Jun 17;20:552. doi: 10.1186/s12913-020-05411-2 (PMC7302358; doi:10.1186/s12913-020-05411-2)
Supplement: Supplementary file 1 — Additional file 1. [file 12913_2020_5411_MOESM1_ESM.pdf]

Supplementary material

1.1 Clinical effectiveness of food consistency modification

Table 1. Aspirations in patients using different levels of consistency modification with xanthan gum (intervention) vs. nil consistency modification (comparator).

Study	Result
**Consistency modification to syrup**	
Leonard 2014	OR=10*; 95%CI: 1.42, 433.98; p=0.01172**
Rofes 2014	p<0.01*
**Consistency modification to custard**	
Rofes 2014	p<0,.01*<br>OR=0.24; 95%CI: 0.08, 0.75; p=0.014<br>RD=-9.17 p.p.; 95%CI: -15.90, -2.43; p=0.0076

* OR was calculated for non-independent samples (http://vassarstats.net/propcorr.html). In the Leonard 2014 study the same sample of patients received two different interventions. In such situation the interpretation of OR is different to classic one, i.e. OR of 10 means that odds of having aspiration were ten times lower for consistency modification compared with nil consistency modification.

** McNemar test.

1.2 Parameters

Probability of dysphagia resolution

The dynamic model attempted to map the natural course of dysphagia in stroke patients. We looked for clinical studies with the longest possible follow-up and dysphagia reporting in subsequent time intervals. Two publications that met these criteria were found (Barer 1989, Smithard 1997). The number of patients experiencing dysphagia on the day of stroke and 7, 28, and 180 days after the stroke onset was reported. Data for 142 patients were included in the analysis.

Table 2. Number of stroke patients diagnosed with dysphagia in time, by Barer 1989, Smithard 1997.

Day	Barer 1989	Smithard 1997	Sum
0	81	61	142
7	25	28	53
28	20	18	38
180	4	2	6

The percentage of patients in whom swallowing disorders are observed in subsequent time intervals was extracted. Data analysis showed that the relationship is the closest to the linear one, if we convert the data to the inverse. Therefore, a model was built and interpolation of results by linear regression was performed. In the following weeks, i.e. from week 27th to 52nd, the proportion of patients diagnosed with dysphagia was modelled using a linear function, approaching zero at the 52nd week of follow-up. The probability of dysphagia symptoms resolution in the following weeks in stroke patients was determined.

Table 3. The natural course of dysphagia in patients after stroke and the weekly probability of resolution of dysphagia symptoms over time.

Time since (weeks)	Percentage of dysphagic patients	Probability of dysphagia resolution	Time since (weeks)	Percentage of dysphagic patients	Probability of dysphagia resolution
0	100.0%	47.9%	27	4.0%	4.1%
1	52.1%	31.3%	28	3.8%	4.3%
2	35.8%	23.9%	29	3.7%	4.5%
3	27.2%	19.3%	30	3.5%	4.7%
4	22.0%	16.1%	31	3.4%	4.9%
5	18.4%	13.9%	32	3.2%	5.1%
6	15.9%	12.2%	33	3.0%	5.4%
7	13.9%	10.9%	34	2.9%	5.7%
8	12.4%	9.8%	35	2.7%	6.1%
9	11.2%	8.9%	36	2.5%	6.5%
10	10.2%	8.2%	37	2.4%	6.9%
11	9.4%	7.6%	38	2.2%	7.4%
12	8.7%	7.0%	39	2.0%	8.0%
13	8.0%	6.6%	40	1.9%	8.7%
14	7.5%	6.2%	41	1.7%	9.6%
15	7.1%	5.8%	42	1.5%	10.6%
16	6.6%	5.5%	43	1.4%	11.9%
17	6.3%	5.2%	44	1.2%	13.5%
18	5.9%	5.0%	45	1.1%	15.6%
19	5.7%	4.7%	46	0.9%	18.4%
20	5.4%	4.5%	47	0.7%	22.6%
21	5.1%	4.3%	48	0.6%	29.2%
22	4.9%	4.1%	49	0.4%	41.2%
23	4.7%	4.0%	50	0.2%	70.0%
24	4.5%	3.8%	51	0.1%	100.0%
25	4.4%	4.1%	52	0.0%	-
26	4.2%	3.9%	-	-	-

Risk of aspiration pneumonia

Non-dysphagic patients

Table 4. Weekly incidence rate of aspiration pneumonia in non-dysphagic patients.

Study ID	Follow-up [months]	n	N	n/N [%]	IR
Sala 1998	6	2	119	1.7	0.0007
Mann 1999	6	2	46	4.3	0.0019
DePippo 1994	2.25	1	57	1.8	0.0020
Reynolds 1998	1	3	33	9.1	0.0238
Teasell 2002	2	0	9	0.0	0.0000
Mean of IR weighted by number of patients in the study					0.0040

Post-stroke mortality

Post stroke mortality was estimated based on stroke incidence by subtype in Poland (calculated as an average weighted by patients' number from three local registries; Jucha

2013, Kozera 2010) and mortality 30-, 90-days and annual mortality in stroke patients by subtype in Poland (Ministerstwo Zdrowia 2018).

Table 5. Incidence of stroke by subtype in Poland.

Stroke subtype	Jucha 2013 (Podkarpackie Province)	Kozera 2010 (Pomeranian Registry)	Kozera 2010 (Świętokrzyskie Province)	Average weighted by the number of patients
Ischemic stroke	80.3%	87.8%	88.9%	87.6%
Primary intracerebral hemorrhage	14.9%	10.3%	9.4%	10.3%
Subarachnoid hemorrhage	4.8%	2.0%	1.8%	2.1%
Number of patients in the study	269	3521	1079	-

Table 6. Annual stroke mortality in Poland in subsequent weeks (Ministerstwo Zdrowia 2018).

Stroke subtype	30-days	90-days	1-year
Ischemic stroke	14.50%	21.50%	30.20%
Primary intracerebral hemorrhage	38.30%	45.10%	52.00%
Subarachnoid hemorrhage	24.30%	27.90%	32.20%
**Average weighted by stroke subtype incidence**	**17.16%**	**24.07%**	**32.50%**
**Parameter**	**Days: 0-30**	**Days: 31-90**	**Days: 91-360**
**Cumulative risk of death in subsequent time intervals**	**17.16%**	**8.34%**	**9.05%**
Number of weeks in subsequent time intervals (out of 52)	4	9	39
Weekly incidence rate	0.0471	0.0097	0.0024
**One-week risk of death in stroke patients in subsequent time intervals**	**4.60%**	**0.96%**	**0.24%**

Table 7. Cumulative mortality and risk of death in subsequent weeks in population of stroke patients based on Jucha 2013.

Time since (weeks)	Cumulative mortality	Death risk	Time since (weeks)	Cumulative mortality	Death risk
0.	0.0%	12.08%	27.	34.8%	0.38%
1.	12.1%	5.44%	28.	35.1%	0.37%
2.	16.9%	3.37%	29.	35.3%	0.36%
3.	19.7%	2.47%	30.	35.5%	0.35%
4.	21.6%	1.97%	31.	35.8%	0.34%
5.	23.2%	1.64%	32.	36.0%	0.33%
6.	24.4%	1.41%	33.	36.2%	0.32%
7.	25.5%	1.24%	34.	36.4%	0.31%
8.	26.4%	1.10%	35.	36.6%	0.31%
9.	27.2%	1.00%	36.	36.8%	0.30%
10.	28.0%	0.91%	37.	37.0%	0.29%
11.	28.6%	0.84%	38.	37.2%	0.29%
12.	29.2%	0.78%	39.	37.4%	0.28%
13.	29.8%	0.73%	40.	37.5%	0.27%
14.	30.3%	0.68%	41.	37.7%	0.27%
15.	30.8%	0.64%	42.	37.9%	0.26%
16.	31.2%	0.61%	43.	38.0%	0.26%
17.	31.6%	0.58%	44.	38.2%	0.25%
18.	32.0%	0.55%	45.	38.3%	0.25%
19.	32.4%	0.52%	46.	38.5%	0.24%

Time since (weeks)	Cumulative mortality	Death risk	Time since (weeks)	Cumulative mortality	Death risk
20.	32.8%	0.50%	47.	38.6%	0.24%
21.	33.1%	0.48%	48.	38.8%	0.23%
22.	33.4%	0.46%	49.	38.9%	0.23%
23.	33.7%	0.44%	50.	39.1%	0.22%
24.	34.0%	0.43%	51.	39.2%	0.22%
25.	34.3%	0.41%	52.	39.3%	0.22%
26.	34.6%	0.40%	-	-	-

Nutilis Clear® average consumption

Table 8. Daily consumption of Nutilis Clear®

Consistency, number of cups per day	Daily Nutilis Clear® consumption [g]	The proportion of patients using a given consistency[1]
syrup, 9	27.00	65
custard, 9	54.00	31
pudding, 9	81.00	5
**weighted mean**	**37.96**	**-**

[1] Due to the data rounding in the publication, the proportion of patients using a given consistency of food sums up to 101%. However, the average was calculated as the weighted average, i.e., e.g. 65 / (65 + 31 + 5), which effects in the proportional distribution of rounding values.

1.3 Systematic review of the health states utilities

A systematic review was carried out to find studies reporting utility of health states. MEDLINE via PubMed and Cochrane Library databases were searched on 02/10/2017. The search strategy is presented in the tables below. In addition, search in the CEA Registry database was carried out (see tables below).

Table 9. Search strategy - utilities.

ID	Query	Cohrane	PubMed
#1	eq 5d OR eq5d OR valuation OR QALY OR utilit* OR "Quality of Life"	73,759	429,651
#2	dysphag*	2,930	24,160
#3	aspiration AND (pneumonia OR pneumonitis)	1,196	144,904
#4	(swallowing OR deglutition) AND (disorder* OR problem*)	1,600	22,050
#5	MeSH descriptor: [Deglutition Disorders] explode all trees	2,386	47,039
#6	#2 or #3 or #4	6,290	70,596
#7	stroke	50,281	275,574
#8	strokes	50,281	280,586
#9	CVA	475	277,228
#10	CVAs	32	247
#11	Apoplexy	395	277,607
#12	(Vascular AND brain) OR Cerebrovascular	17,331	202,018
#13	accident*	13,472	173,510
#14	#12 and #13	7,594	8,343
#15	(brain OR cerebellum) AND infarction	3,391	35,407
#16	brain AND ischemia	4,809	73,171
#17	#7 OR #8 OR #9 OR #10 OR #11 OR #12 OR #14 OR #15 OR #16	85,474	1,459,206
#18	#1 AND #6 AND #17	243	188

Table 10. Keywords for searching the CEA Registry database - utilities.

Keywords	Result
aspiration; Filter: Utility Weights	93
dysphagia; Filter: Utility Weights	21

The review included population of patients with dysphagia, and utility estimates based on population of patients with stroke and dysphagia were preferred over others.

Inclusion criteria:

- reporting the utility of at least one health state out of:
    - dysphagia, if possible, defined as dysphagia with aspiration or without aspiration,
    - aspiration pneumonia in dysphagic patients;
- publication in English or Polish,
- reporting the quality of life for adult patients.

Exclusion criteria:

- in which the aim was to estimate the cost-effectiveness/utility of dysphagia diagnosis/screening.

Systematic review results

A total of 546 abstracts were assessed by two analysts (see the diagram below). 41 records were included for full text analysis and five studies fulfilled the inclusion criteria to our analysis. No other type of research meeting the predefined inclusion criteria has been found. In this, full agreement between analysts was achieved. The list of publications included in the review and excluded are presented in the tables below.

Fig. 1. Selection of studies included in the utilities review.

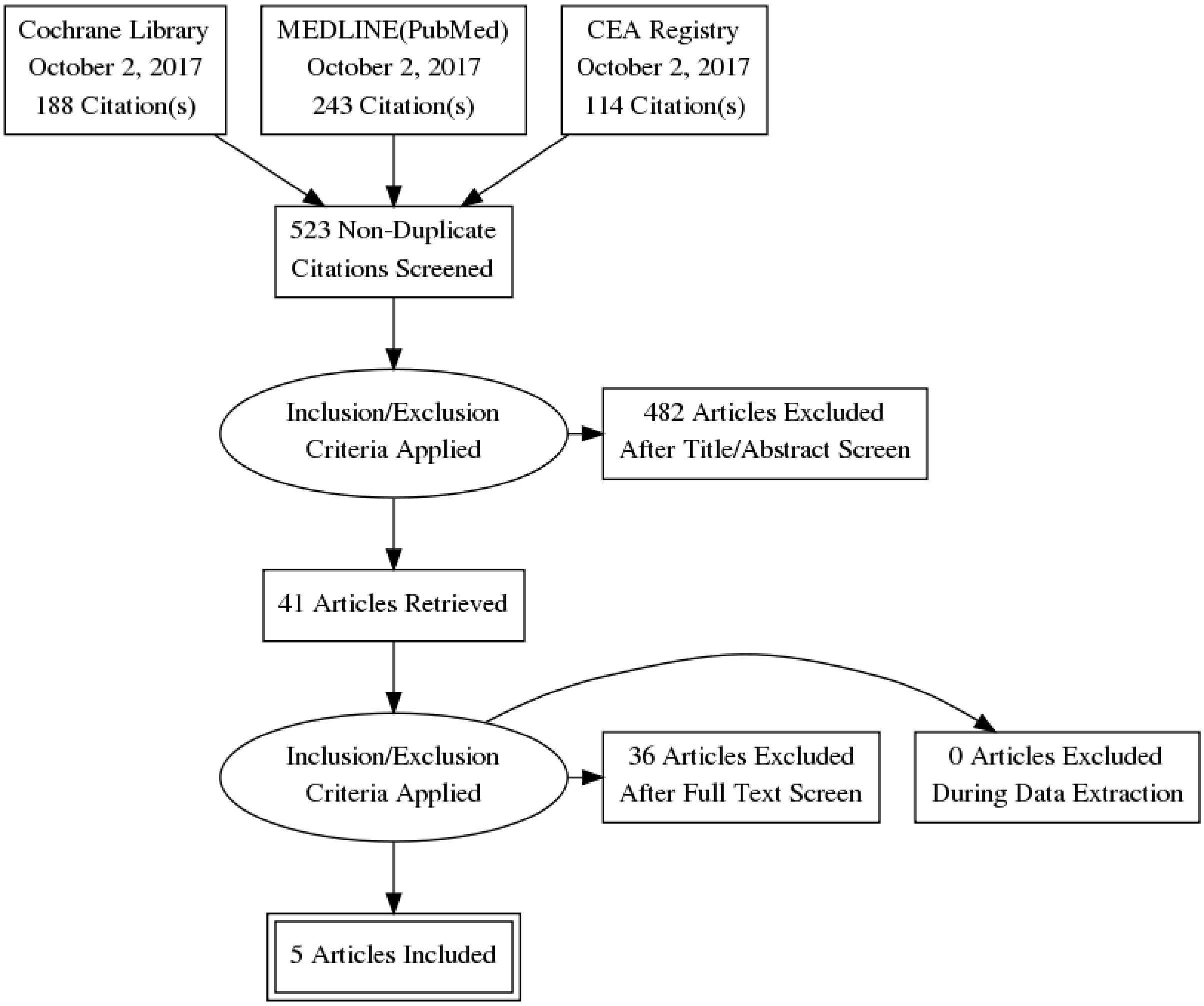

Table 11. List of studies included in the utilities review.

No.	ID	Bibliography
1	Dennis 2005	Dennis MS, Lewis SC, Warlow C, FOOD Trial Collaboration. Effect of timing and method of enteral tube feeding for dysphagic stroke patients (FOOD): a multicentre randomised controlled trial. Lancet. 2005;365(9461):764–72.
2	Mangen 2017	Mangen M-JJ, Huijts SM, Bonten MJM, de Wit GA. The impact of community-acquired pneumonia on the health-related quality-of-life in elderly. BMC Infect Dis [Internet]. 2017.https://www.ncbi.nlm.nih.gov/pmc/articles/PMC5351062/
3	Ramaekers 2011	Ramaekers BLT, Joore MA, Grutters JPC, van den Ende P, Jong J de, Houben R, et al. The impact of late treatment-toxicity on generic health-related quality of life in head and neck cancer patients after radiotherapy. Oral Oncol. 2011 Aug;47(8):768–74.
4	Sturm 2004	Sturm JW, Donnan GA, Dewey HM, Macdonell RAL, Gilligan AK, Srikanth V, et al. Quality of life after stroke: the North East Melbourne Stroke Incidence Study (NEMESIS). Stroke. 2004;35(10):2340–5.

No.	ID	Bibliography
5	Wildi 2004	Wildi SM, Cox MH, Clark LL, Turner R, Hawes RH, Hoffman BJ, et al. Assessment of health state utilities and quality of life in patients with malignant esophageal Dysphagia. Am J Gastroenterol. 2004 Jun;99(6):1044–9.

Table 12. List of studies excluded from the utilities review.

No.	Bibliography	Comment
1	Bath PM, Scutt P, Love J, Clavé P, Cohen D, Dziewas R, et al. Pharyngeal Electrical Stimulation for Treatment of Dysphagia in Subacute Stroke. Stroke. 2016;47(6):1562–70.	No detailed results for the assessed health states.
2	Brandão DM da S, Nascimento JL da S, Vianna LG. Evaluation of functional capacity and quality of life of the elderly after ischemic stroke with and without dysphagia. Revista da Associação Médica Brasileira. 2009;55(6):738–43.	No detailed results for the assessed health states
3	Carlaw C, Finlayson H, Kathleen B, Tiffany V, Caroline M, Coney D, et al. A randomized controlled trial of a water protocol for clients with thin liquid dysphagia. Dysphagia. 2009;24(4):461.	No results. Record in the trials register.
4	Carlaw C, Finlayson H, Beggs K, Visser T, Marcoux C, Coney D, et al. Outcomes of a Pilot Water Protocol Project in a Rehabilitation Setting. Dysphagia. 2012;27(3):297–306.	No detailed results for the assessed health states
5	Cheng IKY, Chan KMK, Wong CS, Cheung RTF. Preliminary evidence of the effects of high-frequency repetitive transcranial magnetic stimulation (rTMS) on swallowing functions in post-stroke individuals with chronic dysphagia. Int J Lang Commun Disord. 2015;50(3):389–96.	No detailed results for the assessed health states
6	Cheng IKY, Chan KMK, Wong C-S, Li LSW, Chiu KMY, Cheung RTF, et al. Neuronavigated high-frequency repetitive transcranial magnetic stimulation for chronic post-stroke dysphagia: A randomized controlled study. J Rehabil Med. 2017;49(6):475–81.	No detailed results for the assessed health states
7	ENOS. Efficacy of nitric oxide, with or without continuing antihypertensive treatment, for management of high blood pressure in acute stroke (ENOS): a partial-factorial randomised controlled trial. The Lancet. 2015;385(9968):617–28.	No detailed results for the assessed health states
8	French B, Thomas LH, Coupe J, McMahon NE, Connell L, Harrison J, et al. Repetitive task training for improving functional ability after stroke. In: Cochrane Database of Systematic Reviews [Internet]. John Wiley & Sons, Ltd; 2016.	No detailed results for the assessed health states
9	Fryer CE, Luker JA, McDonnell MN, Hillier SL. Self-management programmes for quality of life in people with stroke. In: Cochrane Database of Systematic Reviews [Internet]. John Wiley & Sons, Ltd; 2016.	No detailed results for the assessed health states
10	Gilbride JA, Spector S. Nutritional Considerations for the Stroke Patient with Dysphagia. Top Stroke Rehabil. 1996;3(3):51–68.	No data on the quality of life
11	Hong DG, Yoo DH. A comparison of the swallowing function and quality of life by oral intake level in stroke patients with dysphagia. J Phys Ther Sci. 2017;29(9):1552–4.	No detailed results for the assessed health states
12	Ickenstein GW, Riecker A, Höhlig C, Müller R, Becker U, Reichmann H, et al. Pneumonia and in-hospital mortality in the context of neurogenic oropharyngeal dysphagia (NOD) in stroke and a new NOD step-wise concept. J Neurol. 2010;257(9):1492–9.	No detailed results for the assessed health states
13	Kang Y, Lee H-S, Paik N-J, Kim W-S, Yang M. Evaluation of enteral formulas for nutrition, health, and quality of life among stroke patients. Nutr Res Pract. 2010;4(5):393–9.	No detailed results for the assessed health states
14	Kang J-H, Park R-Y, Lee S-J, Kim J-Y, Yoon S-R, Jung K-I. The Effect of Bedside Exercise Program on Stroke Patients with Dysphagia. Ann Rehabil Med. 2012;36(4):512–20.	No detailed results for the assessed health states
15	Khaldoun E, Woisard V, Verin E. Validation in French of the SWAL-QOL scale in patients with oropharyngeal dysphagia. Gastroenterol Clin Biol. 2009;33(3):167–71.	No detailed results for the assessed health states
16	Klinke ME, Wilson ME, Hafsteinsdóttir TB, Jónsdóttir H. Recognizing new perspectives in eating difficulties following stroke: a concept analysis. Disabil Rehabil. 2013;35(17):1491–500.	No data on the quality of life
17	Laver KE, Schoene D, Crotty M, George S, Lannin NA, Sherrington C. Telerehabilitation services for stroke. In: Cochrane Database of Systematic Reviews [Internet]. John Wiley & Sons, Ltd; 2013.	No detailed results for the assessed health states
18	Lemmens J, Bours GJJW, Limburg M, Beurskens AJHM. The feasibility and test–retest reliability of the Dutch Swal-Qol adapted interview version for dysphagic patients with communicative and/or cognitive problems. Qual Life Res. 2013;22(4):891–5.	No detailed results for the assessed health states

No.	Bibliography	Comment
19	McCurtin A, Healy C, Kelly L, Murphy F, Ryan J, Walsh J. Plugging the patient evidence gap: what patients with swallowing disorders post-stroke say about thickened liquids. Int J Lang Commun Disord. 2017 Jun 16; [Internet].	No data on the quality of life
20	Perry A, Lee SH, Cotton S, Kennedy C. Therapeutic exercises for affecting post-treatment swallowing in people treated for advanced-stage head and neck cancers. In: Cochrane Database of Systematic Reviews [Internet]. John Wiley & Sons, Ltd; 2016.	No detailed results for the assessed health states
21	Pontes ÉS, Amaral AK de FJ do, Rêgo FLC do, Azevedo EHM, Silva POC, Pontes ÉS, et al. Quality of life in swallowing of the elderly patients affected by stroke. Arquivos de Gastroenterologia. 2017;54(1):27–32.	No detailed results for the assessed health states
22	Pooyania S, Vandurme L, Daun R, Buchel C. Effects of a Free Water Protocol on Inpatients in a Neuro-Rehabilitation Setting. Open Journal of Therapy and Rehabilitation. 2015;03(04):132–8.	No data on the quality of life
23	Qu S-H, Li M, Liang J-P, Su Z-Z, Chen S-Q, He X-G. Laryngotracheal Closure and Cricopharyngeal Myotomy for Intractable Aspiration and Dysphagia Secondary to Cerebrovascular Accident. ORL. 2009;71(6):299–304.	No data on the quality of life
24	Robbins J, Kays SA, Gangnon RE, Hind JA, Hewitt AL, Gentry LR, et al. The effects of lingual exercise in stroke patients with dysphagia. Arch Phys Med Rehabil. 2007;88(2):150–8.	No detailed results for the assessed health states
25	Rodrigue N, Côté R, Kirsch C, Germain C, Couturier C, Fraser R. Meeting the nutritional needs of patients with severe dysphagia following a stroke: an interdisciplinary approach. Axone. 2002;23(3):31–7.	No full text
26	Sanders H, Newall S, Norton B, Holmes GT. Gastrostomy feeding in the elderly after acute dysphasgic stroke. J Nutr Health Aging. 2000;4(1):58–60.	No full text
27	Schimmel M, Ono T, Lam OLT, Müller F. Oro-facial impairment in stroke patients. J Oral Rehabil. 2017;44(4):313–26.	No data on the quality of life
28	Smithard D, Dias R. Subjective swallowing difficulties following stroke: a questionnaire survey. Clin Rehabil. 1997;11(4):350–2.	No data on the quality of life
29	Swan K, Speyer R, Heijnen BJ, Wagg B, Cordier R. Living with oropharyngeal dysphagia: effects of bolus modification on health-related quality of life--a systematic review. Qual Life Res. 2015;24(10):2447–56.	No detailed results for the assessed health states
30	Tran P, Mannen J. Improving oral healthcare: improving the quality of life for patients after a stroke. Spec Care Dentist. 2009;29(5):218–21.	No data on the quality of life
31	Verin E, Maltete D, Ouahchi Y, Marie J-P, Hannequin D, Massardier EG, et al. Submental sensitive transcutaneous electrical stimulation (SSTES) at home in neurogenic oropharyngeal dysphagia: A pilot study. Annals of Physical and Rehabilitation Medicine. 2011;54(6):366–75.	No detailed results for the assessed health states
32	Xia W, Zheng C, Lei Q, Tang Z, Hua Q, Zhang Y, et al. Treatment of post-stroke dysphagia by vitalstim therapy coupled with conventional swallowing training. J Huazhong Univ Sci Technol Med Sci. 2011;31(1):73–6.	No detailed results for the assessed health states
33	Xia W, Zheng C, Zhu S, Tang Z. Does the addition of specific acupuncture to standard swallowing training improve outcomes in patients with dysphagia after stroke? a randomized controlled trial. Clin Rehabil. 2016;30(3):237–46.	No detailed results for the assessed health states
34	Xiao Y, Luo M, Wang J, Luo H. Inspiratory muscle training for the recovery of function after stroke. Cochrane Database Syst Rev. 2012;(5):CD009360	No detailed results for the assessed health states
35	Zhang M, Tao T, Zhang Z-B, Zhu X, Fan W-G, Pu L-J, et al. Effectiveness of Neuromuscular Electrical Stimulation on Patients With Dysphagia With Medullary Infarction. Arch Phys Med Rehabil. 2016;97(3):355–62.	No data on the quality of life.
36	Zhang C, Bian J, Meng Z, Meng L, Ren X, Wang Z, et al. Tongguan Liqiao acupuncture therapy improves dysphagia after brainstem stroke. Neural Regen Res. 2016;11(2):285–91.	No detailed results for the assessed health states

1.4 Sensitivity analysis

Static model

One-way sensitivity analysis

Sensitivity analysis involved parameters with the greatest uncertainty testing (risk of pneumonia and the utilities of health states).

Table 13. List of parameters, one-way sensitivity analysis - static model.

Parameter	Value/range	Data source, comment
The risk of aspiration pneumonia in patients without aspiration	min: 6.08%; max: 20.05%	DePippo 1994, own estimation of 95%CI based on the beta distribution
The risk of aspiration pneumonia in patients with aspiration	min: 0.05%; max: 6.37%	DePippo 1994, own estimation of 95%CI based on the beta distribution
The utility of aspiration	min: 0.288; max: 0.388	Ramaekers 2011, Wildi 2004; own estimation of 95%CI
Decrease in utility caused by the occurrence of aspiration pneumonia	min: 0.11; max: 0.16	Mangen 2017

Scenario sensitivity analysis

Different consumption of Nutilis Clear®

In this scenario, we assumed a different consumption of Nutilis Clear®. According to GUSS score, the result of 10-14 points corresponds to medium-severity dysphagia with moderate aspiration risk. While according to the Nutilis Clear® leaflet in medium-severity dysphagia, it is recommended to use custard consistency, which corresponds to the 54g product per day, i.e. 2.16 packs per week.

Different costs of aspiration pneumonia treatment

In this scenario, we assumed that the costs of aspiration pneumonia treatment is lower and equals the costs of pneumonia without complications treatment (according to NFZ data). The cost of treatment of aspiration pneumonia in this scenario of sensitivity analysis was therefore assumed to PLN 1,300.

Probabilistic sensitivity analysis

Parameters used in the calculations were estimated based on random samples, so their value is subject to statistical error. The impact of these errors (occurring simultaneously for all estimated model parameters) was examined using probabilistic sensitivity analysis (PSA) using Monte Carlo simulation approach. The risk of aspiration pneumonia and the utilities were randomized using beta distribution to guarantee the parameters remain positive.

Table 14. Static model PSA – parameters distribution.

Type	Parmeter	Parameter distribution
Clinical parameters	p(pneumonia in aspirating)	beta
	p(pneumonia in non-aspirating)	beta
Utilities	U(aspirating)	beta
	U(non-aspirating)	beta
	U(pneumonia)	beta

Dynamic model

One-way sensitivity analysis

Sensitivity analysis involved parameters with the greatest uncertainty testing (risk of pneumonia, aspiration pneumonia health state utilities and treatment costs).

Table 15. List of parameters used in the one-way sensitivity analysis – dynamic model.

Parameter	Value/range	Data source, comment
Clinical parameters		
risk of aspiration pneumonia in stroke patients without dysphagia [%]	min: 0.00; max: 2.56	own estimation of 95%CI based on: Sala 1998, Mann 1999, DePippo 1994, Reynolds 1998, Teasell 2002
relative risk of aspiration pneumonia in patients with dysphagia but without aspiration [p.p.]	min: 1.03; max: 7.42	95%CI; Pikus 2003
relative risk of aspiration pneumonia in patients with dysphagia and aspiration [p.p.]	min: 3.28; max: 20.85	95%CI; Pikus 2003
relative risk of death in stroke patients with aspiration pneumonia [p.p.]	min: 2.44; max: 3.66	95%CI; Katzan 2003
Utilities		
no dysphagia	min: 0.42; max: 0.52	95%CI; Sturm 2004
dysphagia with aspiration	min: 0.288; max: 0.388	own estimation of 95%CI based on: Dennis 2005, Ramaekers 2011, Wildi 2004
aspiration pneumonia (decrement)	min: 0.10; max: 0.15	Mangen 2017

Scenario sensitivity analysis

Positive prediction value of the GUSS test

In this scenario, we assumed a false positive diagnosis is possible during the assessment of the patient's condition with the GUSS screening test. Hence, in some patients Nutilis Clear® will be used unnecessarily.

The percentage of patients with a false positive diagnosis was estimated based on the results of Trapl 2007 and Warnecke 2017 studies. In both studies, the diagnosis of aspiration and dysphagia was carried out using instrumental methods (FPS, FEES) and GUSS screening test. The use of the GUSS test may result in increased percentage of patients with the diagnosis of aspiration in comparison with the use of instrumental methods.

Table 16. The structure of diagnoses using the GUSS test and instrumental methods based on Trapl 2007 and Warnecke 2017 (PPV = positive predictive value).

Study/group	GUSS+/FEES+	GUSS+/FEES-	Sum	PPV
Trapl 2007/ I	13	3	16	81.25%
Trapl 2007 / II	14	5	19	73.68%
Warnecke 2017	55	19	74	74.32%
			weighted mean	75.23%

Cost-effectiveness analysis

The scenario was conducted in agreement with EUnetHTA guidelines for health economic evaluations (EUnetHTA EA 2015). The scenario assumes that the effectiveness of Nutilis Clear® stems only from the patients' years of life gained (LYG).

Lifetime analysis horizon

Further survival (after 1st year) of stroke patients in Poland was estimated based on Boysen 2009 and the average age of patients on the day of stroke occurrence based on Jucha 2013.

Risk of death in stroke patients based on Jucha 2013

In this scenario, we assumed the risk of death in stroke patients as observed in Jucha 2013 study. The study included 269 stroke patients (the first in life or recurrent), hospitalized in the Regional Hospital in Krosno in 2003/2004. Results were standardized to the European population, in terms of age and gender structure. Mortality was assessed after 30, 90, 180 days and a year after the stroke. The data on cumulative mortality over subsequent time intervals was interpolated using a logarithmic function. Very high reproduction of experimental points was obtained ($y = 0.069 \cdot \ln(x) + 0.1208$, where y was cumulative mortality [%] and x was the time in weeks; R2 = 0.9947). Based on the mortality curve, the risk of death in subsequent weeks after stroke were determined in a one-year period. See table Table 7.

The lack of monitoring

The scenario assumes that monitoring will take place in a natural way and will not involve additional costs for the public payer.

Different costs of aspiration pneumonia treatment

Check assumptions made for static model (above).

Different consumption of Nutilis Clear®

Check assumptions made for static model (above).

Probabilistic sensitivity analysis

Parameters used in the calculations were estimated based on random samples, so their value is subject to statistical error. The impact of these errors (occurring simultaneously for all estimated model parameters) was examined using probabilistic sensitivity analysis (PSA) using Monte Carlo simulation approach. The risk of aspiration pneumonia and the utilities were randomized using beta distribution to limit the results to positive values.

Table 17. Dynamic model PSA – parameters distribution.

Type	Parameter	Parameter distribution
Clinical parameters	p(pneumonia in no dysphagia)	beta
	RR pneumonia (dysphag no aspir.)	normal
	RR pneumonia (dysphag and aspir.)	normal
	RR mortality (pneumonia)	normal
	p(dysphagia deterioration)	uniform
Costs	C(BSC)	uniform
	C(Nutilis)	uniform
	C(Pneumonia)	uniform
	C(No dysphagia)	uniform
	C(Monitoring)	uniform
Utilities	U(No dysphagia)	beta
	U(Dysphagia and aspiration)	beta
	U(Dysphagia, no aspiration)	beta
	U(Pneumonia)	beta

1.5 Results

Detailed results of modeling are presented below. All calculations were performed form the public payer perspective. Results from joint perspective are very similar due to marginal patient's copayment for therapy.

Table 18. Basic analyses and scenario sensitivity analyses results.

Scenario	Static model [PLN]	Dynamic model [PLN]
Basic	21,387	20,977
Not ideal PPV by GUSS test	-	35,764
CEA	-	77,389
No monitoring	-	14,318
Pneumonia costs	23,283	23,554
Mortality based on Jucha study	-	21,899
Lifetime horizon	-	20,671
Different Nutilis consumption	32,896	33,164

Fig. 2. PSA results (dynamic model)

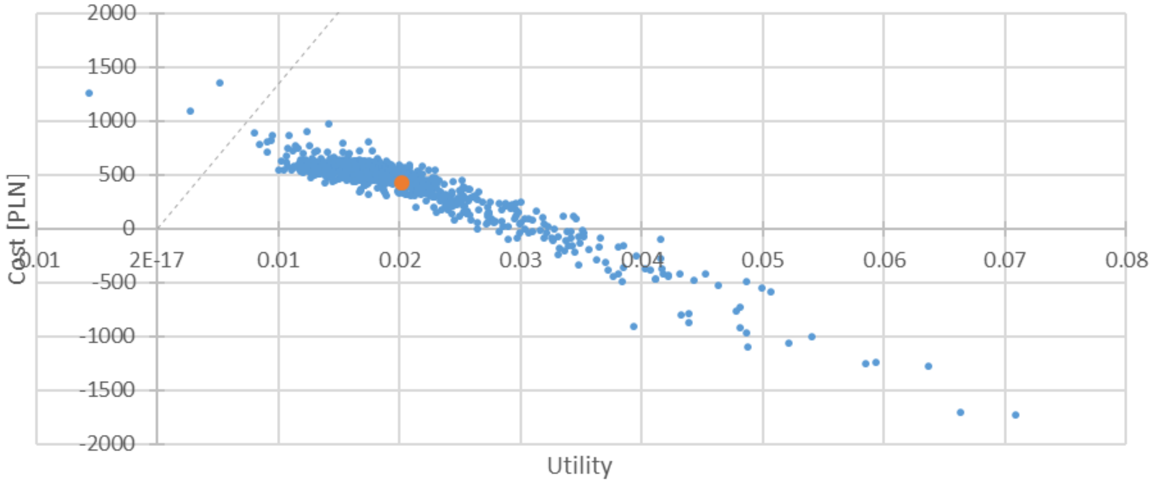

Fig. 3. Cost-effectiveness acceptability curve (dynamic model)

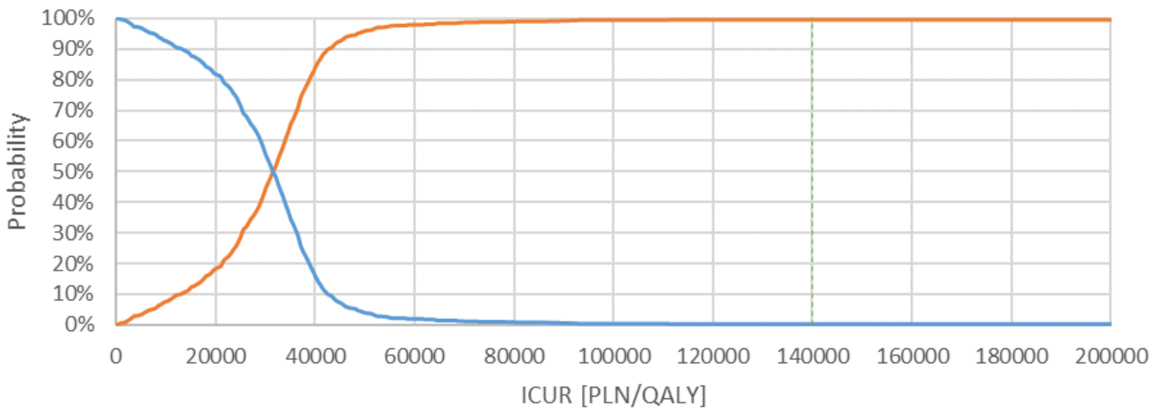

Fig. 4. PSA results (static model)

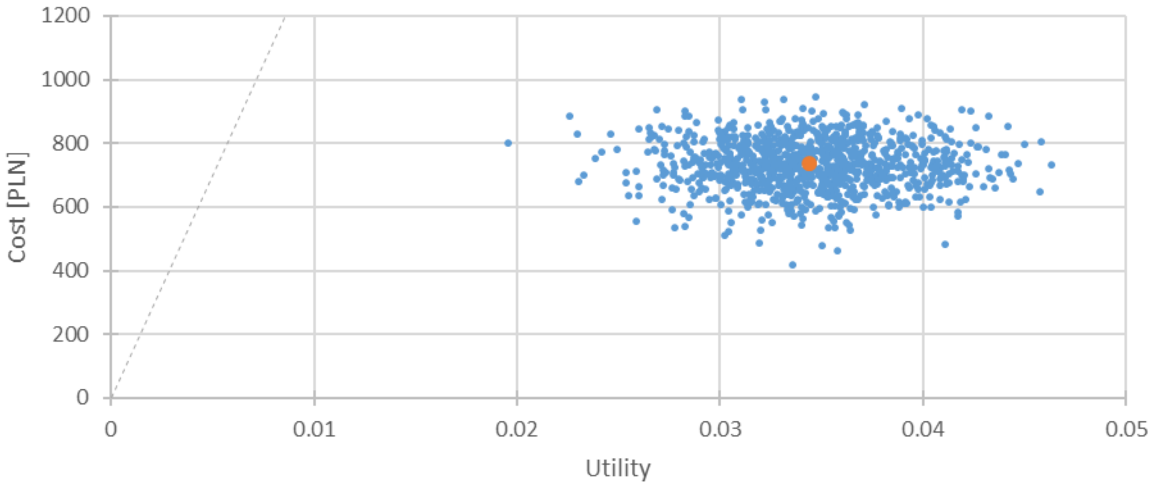

Fig. 5. Cost-effectiveness acceptability curve (static model)

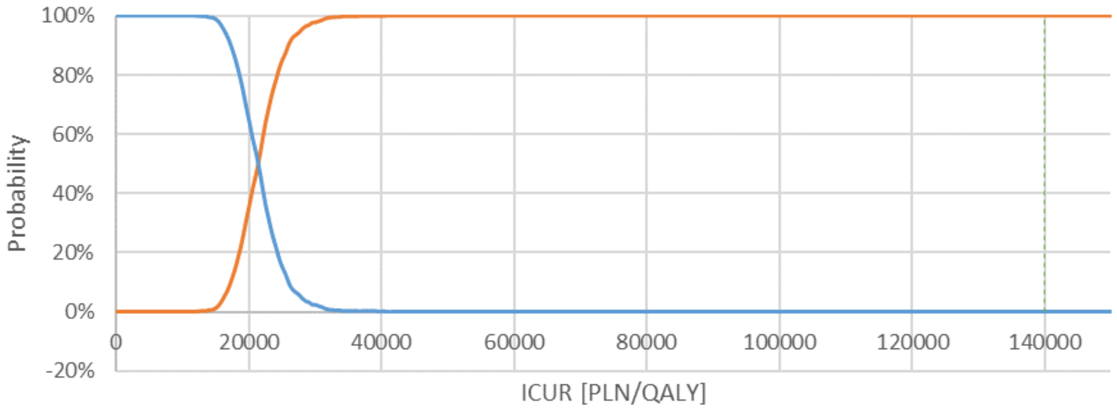

1.6 Additional references

Boysen G, Marott JL, Grønbæk M, Hassanpour H, Truelsen T. Long-Term Survival after Stroke: 30 Years of Follow-Up in a Cohort, the Copenhagen City Heart Study. NED. 2009;33(3):254–60.
